# Diagnose earlier, live longer? The impact of cervical and breast cancer screening on life span

**DOI:** 10.1371/journal.pone.0270347

**Published:** 2022-07-20

**Authors:** Zhenjie Yang, Juan Liu, Qing Wang

**Affiliations:** 1 Faculty of Humanities and Social Sciences, Macao Polytechnic University, Macao, People’s Republic of China; 2 School of Public Administration, South China Agricultural University, Guangzhou, People’s Republic of China; 3 School of Public Health, Shandong University, Jinan, People’s Republic of China; Xiamen University, CHINA

## Abstract

Cancer has become a leading cause of death and aroused the cancer scare. Breast and cervical cancer are two main health threats for women. In order to reduce mortality through early detection and early treatment, cancer screening has been widely recommended and applied for breast and cervical cancer detection and prevention. However, the benefit of cancer screening has been a controversial issue for the recent decades. The Chinese government has launched a free screening program on breast and cervical cancer for women since 2009. There is lack of strong data and sufficient information, however, to examine the effect of breast and cervical cancer screening. A Difference-in-Difference model estimated by Cox proportional hazard estimation was applied to evaluate the effects of breast and cervical cancer screening using data from Nown County Cancer Registry between the year 2009 and 2013. Based on the case study in a county of central China, this study found that the screening program reduced the risk of death, but found the lion’s share for the benefit has been mainly due to the cervical cancer screening rather breast cancer screening, which may be related to the difference between early detection screening and preventive screening. Our results suggest sufficient funding and better education of related cancer knowledge will be meaningful measures for the prevention and treatment of breast and cervical cancer.

## Introduction

Cancer has become a leading cause of death and aroused the cancer scare due to the dramatically increased incidence and death rate [1, 2]. Breast cancer and cervical cancer are two of three sharply increased cancers for women, especially in developing countries. Cervical cancer increased 4.1% from 2007 to 2011 while breast cancer did 3.9% from 2000 to 2011. The incidence and death rate of breast cancer and cervical cancer in China was much higher than developed countries. For instance, cervical cancer incidence for women in China was 15.2 per 100, 000 while it was only 6.5 per 100, 000 in the United States; the cervical case in China amounted to 28% of half million cases of the whole world and 50% of Asia, and the death number amounted to 1/4-1/3 of Asia [3]. Cancer screening has been globally adopted and recommended as the necessary measure for cancer prevention and treatment. The Chinese government also launched the cancer screening in breast and cervix for women since 2009. Later, this program was listed in the Ten-Year Outline for the development of women in China, with the intention to reduce mortality through early detection and treatment [4]. Though cancer screening has been considered as a popular measure for cancer detection and prevention, its effect in improving life length varied among different countries, and is still doubtful as the existing literature is mainly descriptive and correlation analysis. Does the breast and cervical cancer screening extend patients’ life span? Does the cancer screening have different effects in urban and rural areas? Does the breast and cervical cancer screening have different effects in extending patients’ lives? Are there different effects of the detection and treatment between rural women who take farming and those who do not take farming?

Cancer screening program has been widely accepted with the intention to detect and diagnose patients as soon as possible. However, the effect of breast and cervical cancer screening programs has been a contested issue since a series of factors, such as the screening age scope and population, technology, influenced the result [5–7]. On the one hand, some studies showed that breast and cervical cancer incidence and mortality has decreased significantly over the past four decades in lots of well-developed countries with the well-designed cancer screening institution, which has been attributed to widespread application of screening tests [8]. Effective screening is available to detect precancerous lesions or early-stage breast or cervical cancer [9], and early detection allows for more treatment options with better health-related outcomes, improves survival rates and lowers healthcare costs [6, 10–12]. On the other hand, the result did not produce significant effect though some researches touched upon the puzzle whether the breast cancer screening reduced the breast cancer mortality [13–17], particularly for women below 50 years old [7]. Some scholars pointed out the necessity and strategies to balance the risk and benefit of mammography screening programs [18]. The experiences of developed countries exhibited that density and quality of screening and access to the health care influenced the result [19–21]. It was found that age and parity influence the performance of visual tests for cervical cancer screening [22]. It normally took several years to see the effect of screening programs. In well developed countries (e.g., Nordic countries, Canada and the UK), the decline in breast and cervical cancer prevalence can be observed in 5–7 years from initiation [23–27]. Besides, researchers also raised the worry that early detection and treatment may bring in psychological fear and over-diagnosis, which produced the counter result and caused the unnecessary death increase [28–31]. Scholars also raised the concern that the reduction in cancer mortality may be attributed to the advance of treatment and vaccination rather than the screening program [32, 33]. Scholars also realized the influence of COVID-19 over cancer screening [34].

Another issue is the research method. Randomized controlled trial (RCT) was the most popular method in cancer studies besides the cascade analysis and case studies [35, 36]. However, as it is high-cost experiment and targets at the homogeneous samples, RCT still encountered two problems: one is the lack of sufficient reliable data; the other one is that RCT may be not as appropriate for the homogeneous data. It is necessary to evaluate the effect of cancer screening scientifically with more sufficient reliable data and appropriate methods, such as internationally recognized high-quality data and quasi-experiment method with less cost.

Little information is known about the effect of the screening program in breast and cervical cancer in China, since the Chinese government has launched a free screening program on breast and cervical cancer for women since 2009. This study applied a Difference-in-Difference model estimated by Cox proportional hazard estimation to evaluate the effects of breast and cervical cancer screening using data from Nown County (a pseudonyms name) Cancer Registry between the year 2006 and 2016. Based on the case study in a county of central China, this study found that the screening program reduced the risk of death, but the lion’s share for the benefit has been mainly due to the cervical cancer screening rather breast cancer screening, which may be related to the difference between early detection screening and preventive screening. This research also found that the screening program produced better effect for those patients who taking non-farming jobs than those taking farming jobs, and there was no obvious difference in the screening effect of reducing mortality between rural and urban areas.

The rest of the paper was organized as follows: it first introduces the policy background and the empirical model; Then, it describes the data and presents summary statistics; Next, it reports the main results and validity tests, conducts the robustness tests, placebo test and the heterogeneity analyses; Finally, it summaries and concludes.

## The policy background and empirical model

Since 2012, Nown county in central China began to implement the **free** cytology-based cervical and breast cancer screening program. All the women aged 35–64 years, who have lived in Nown county for more than one year and were never diagnosed with cervical or breast cancer, were eligible to the cancer screening program. Note that cancer examination is voluntary, namely, the eligible women could choose not to participate in the program. The whole screening consists of three components. First, the village/community doctors were responsible for informing eligible women about the cancer screening program. Second, the township/sub-district health center and the County Maternity and Child Health Hospital were in charge of the tests. Third, the County Health Bureau took the charge of quality control. S1 Appendix presents the details of the cancer screening program. In addition, a follow-up survey after the diagnosis was conducted until now. Note that this survey only contains the women with a cancer no matter whether or not they were covered by the screening program. Roughly, three quarters of the patients were still alive in the last follow-up, whose final life span were unobserved.

**Fig 1:**
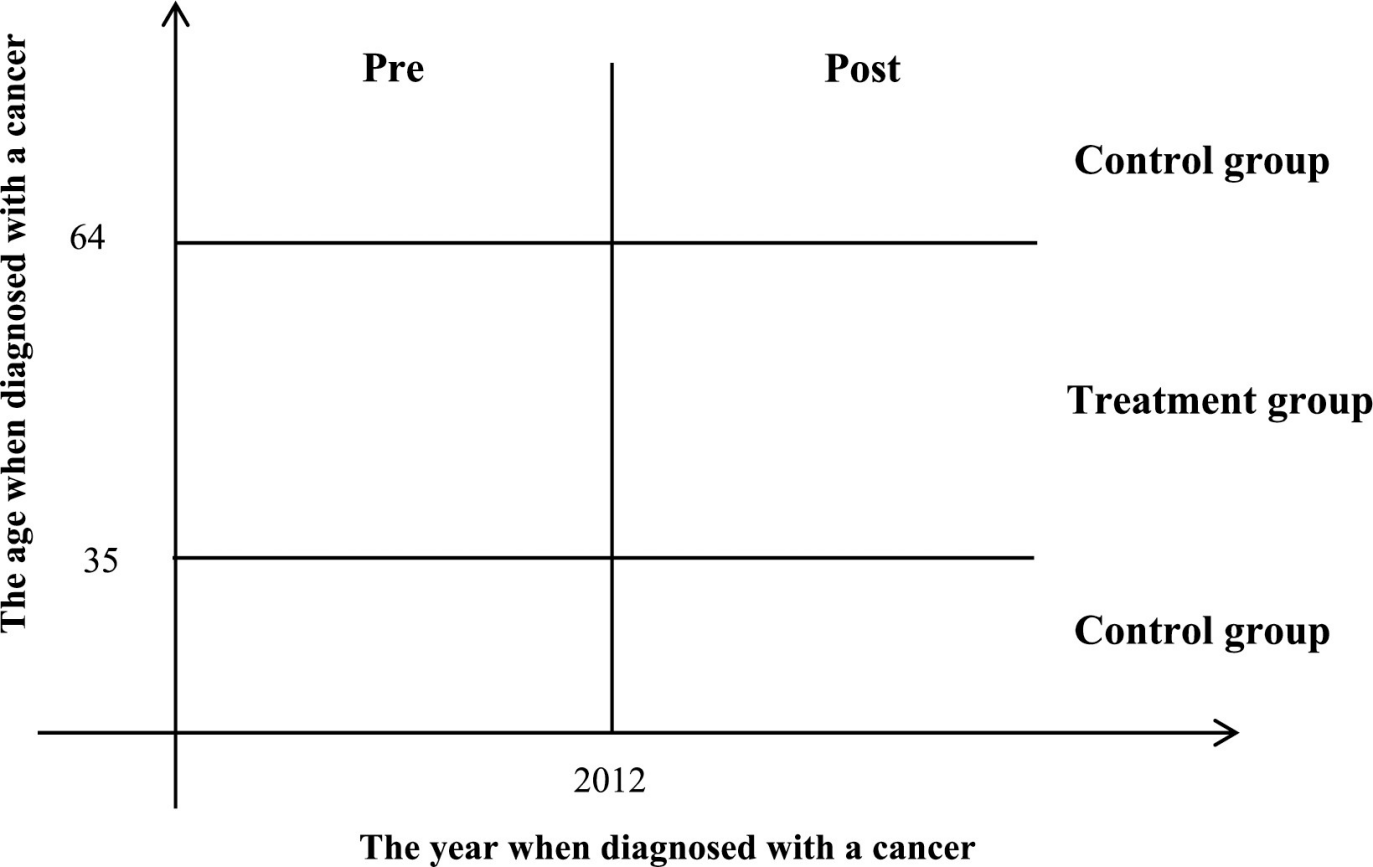
The DiD model setting up.

According to the design of the policy, only the women aged 35–64 were eligible to the screening program, while the other women (aged below 35 or above 64) were not. Thus, the women in the two different age groups naturally comprise the treatment and control groups, respectively. Given this, we intend to examine the causal effect of cervical and breast cancer screening on life span through the Difference-in-Differences (DiD) model. The DiD model setting up can be roughly illustrated by Fig 1. Note that the data we are using is a pooling cross-sectional data and only the women who are diagnosed with a cancer are included in the data, which will be described in detail in the next section.

In addition, the fact that life span is censored from right implies that we need to combine DiD model with Cox proportional hazard (PH) model, namely a Cox PH DiD model. Specifically, we consider the following hazard regression equation:

$$\lambda (t)=\lambda_{0}(t)\exp\left(\beta_{1}{POST}_{j}+\beta_{2}{Treat}_{i}+\beta_{3}({POST}_{j}∙{Treat}_{i})+X_{ij}^{\prime }\delta +\varepsilon_{ij}\right),$$ (M1)

where *t* is the **time duration** from diagnosis to death or to the close of the study for patient *i* who was diagnosed with a breast or cervical cancer in year *j* and still alive up to now or till the date of loss of follow-up; *λ*(*t*) is the hazard rate, namely, the probability of the event that an individual die from cervical or breast cancer in time *t*, conditional on that the individuals suffering from cancer through period *t*. *λ*_0_(*t*) represents the baseline hazard and depends on the time *t* alone, which we do not care about. POST equals 1 if cervical or breast cancer examination was implemented since 2012, and 0 otherwise; Treat equals 1 if a patient aged between 35 and 64 in the diagnosis time, and 0 otherwise; X is a vector of control variables including age of incidence and its square, residential address (urban or rural areas), marital status, and occupations; *ε* is the error term.

In this study, the coefficient *β*_3_ is the primary interest, which measures the causal effect of cervical and breast cancer screening on hazard rate of dying conditional on the other covariates. Specifically,

$$\begin{array}{l} \frac{\lambda (t|X_{ij},\;{POST}_{j}=1,\;{Treat}_{i}=1)/\lambda (t|X_{ij},\;{POST}_{j}=0,\;{Treat}_{i}=1)}{\lambda (t|X_{ij},\;{POST}_{j}=1,\;{Treat}_{i}=0)/\lambda (t|X_{ij},\;{POST}_{j}=0,\;{Treat}_{i}=0)}\\ \;=\frac{exp(\beta_{1}+\beta_{2}+\beta_{3}+X_{ij}^{\prime }\delta )/exp(\beta_{2}+X_{ij}^{\prime }\delta )}{exp(\beta_{1}+X_{ij}^{\prime }\delta )/exp(X_{ij}^{\prime }\delta )}=\frac{exp(\beta_{1}+\beta_{3})}{exp(\beta_{1})}=\exp\left(\beta_{3}\right),\; \end{array}$$

where the first row of the above equation is the DiD in the context of the hazard rate. The numerate is the pre-post difference in terms of the ratio for the treatment group, while the denominate is the pre-post difference in terms of the ratio for the control group. In the Cox PH model, the hazard rate is factored into a baseline hazard and an exponential function of explanatory variables. *The above equation suggests the DiD estimate for the impact of the cancer screening program on the* ***hazard rate*** *of dying is exp*(*β*_3_)−1. That is, patients being eligible to the cancer screening are *exp*(*β*_3_)−1 more likely to die in the time *t*, compared to the case if they are not eligible to the cancer screening. *If screening implies early diagnosis and early treatment*, *and thus live longer*, *we can expect β*_3_ *to be negative*. *On the contrary*, *if the cancer happens inevitably*, *early diagnosis may make patients sad*, *anxious and depressed*, *consequently*, *the patients may die quicker*. *In this case*, *β*_3_ *should be positive*.

Here, we need to note that because the screening is voluntary rather than compulsory. It is possible that the women who are more confident about their health are less likely to participate in the screening program, or the women who have more health knowledge are more likely to participate in the screening program and also, they may take better care of themselves once they were diagnosed with a cancer. These two sample selection issues may cause different bias: the first one leads to an underestimate while the second leads to an overestimate. If we have the data of health knowledge and subjective evaluation of health status before the diagnosis, we may control for this problem. Unfortunately, due to the data limitation, we cannot do this in this paper.

In the next section, we will see that there are six and five years before and after the screening program, respectively. However, Model M1 assumes that the baseline hazard rate is constant in both pre- and post-treatment periods, which is a little restrictive. Thus, we augment Model M1 by replacing the dummy for post-treatment with a set of dummies for the years of incidence:

$$\lambda (t)=\lambda_{0}(t)\exp\left(\sum_{j=2007}^{2016}{(\beta }_{1}^{j}∙D_{j})+\beta_{2}{Treat}_{i}+\beta_{3}({POST}_{j}∙{Treat}_{i})+X_{ij}^{\prime }\delta +\varepsilon \right),$$ (M2)

where {D_*j*_} is a set of dummy variables indicating in what year the patient was diagnosed with a cancer. Compared to Model M1, Model M2 is more flexible and thus it is the preferred specification.

## Data and summary statistics

This study used data from Nown county Cancer Registry, one of National Central Cancer Registry (NCCR) of China recognized and data-quality-compliant registries. The NCCR program was launched and funded by the Ministry of Health of China in 2008 to promote standardizing cancer data and improving data quality of local registries [37]. Local registries distributed in different regions, are responsible for the collection of cancer patients’ medical information (personal information, incidence, mortality and survival data, type and grade of cancer, etc.) from hospitals, community health centers, urban and rural health insurance programs, etc. NCCR is responsible for guiding, supporting and funding local registries, in particular evaluating and monitoring their data quality. Although there were 234 local registries scattered within 31 provinces, autonomous regions and municipalities till 2014, only 177 registries’ data complied with the data-quality criteria of the internationally recognized standard—Guidelines of Chinese Cancer Registration and International Agency for Research on Cancer/International Association of Cancer Registries (IARC/IACR) [38]. Nown county registry is one of those registries recognized by NCCR in data quality. This research has been reviewed and approved by the Ethics Committee of School of Public Administration, South China Agricultural University, and does not involve ethical relevant information. Identities of patients have been completely anonymous, and there is no possible legal, social or economic risk to our research subjects.

The raw data from Nown county Cancer Registry consists of 15,955 patients including both males and females with different types of cancer. Of these patients, 2,201 women were diagnosed with breast or cervical cancer. Because the different cancers may not be comparable, while the screening program only covers the breast and cervical cancer, we exclude those patients with any cancers other than these two cancers. Among the women with breast and cervical cancers, 1,065 patients were failed to follow up, and extra 58 patients have missing values in age, occupation, residential address or any other variables. Finally, the sample used in this paper contains 1,078 observations. For this data set, we need to make two notes. First, only the women who have a cancer were included. That is those who participated the screening program but do not have a cancer are not in the sample. Second, for each person, we have only one observation which records the year when she was diagnosed with a cancer, whether she has died by the year, and if yes the year when she was deceased.

Table 1 presents the summary statistics for the treatment and control groups, separately, as well as pre- and post-treatment periods, separately. Let us look at the pre-treatment (Panel A) first. On average, the time duration since the diagnose for the control group is 819 days, while it is 395 days longer for the treatment group. Given the fact that 95% of the control group have died but the corresponding proportion is 80%, the average life span of the treatment should be longer than 395 days. However, this is not surprising, because in general the control group is diagnosed at a much older age than the treatment group: 66 vs. 50. In the control group, one thirds of patients have breast cancer and two thirds have cervical cancer in the control group. In contrast, 58% of patients have the breast cancer and 42% have the cervical cancer in the treatment group. This difference may be due to the control group is older when they were diagnosed with a cancer. In terms of residential address, marriage status and occupation, there is only trivial difference between the control and treatment groups, though the difference in the marriage status is statistically significant. The pre-treatment periods include six years, but in the first four years, there are very limited observations especially in the control groups–only 18% of the patients in the control group were diagnosed over the period 2006–2009.

**Table 1:** Summary statistics.

	Control group		Treatment group		Diff.
	Mean	S.D	Mean	S.D	(3)-(1)
Variables	(1)	(2)	(3)	(4)	(5)
**Panel A: Pre-treatment**					
Duration time from diagnose to death or the last follow-up (days)	819	629	1,214	1,033	395**
Died	0.95	0.22	0.80	0.40	-0.15
Age of incidence	66.06	15.46	49.83	7.66	-16.00**
Breast cancer	0.33	0.47	0.58	0.50	-0.25**
Cervical cancer	0.67	0.47	0.42	0.50	0.25**
Rural area	0.54	0.5	0.52	0.50	-0.02
Married but not widowed	0.96	0.2	1.00	0.00	0.04**
Peasant	0.93	0.25	0.89	0.32	-0.05
Year of incidence					
2006	0.01	0.11	0.10	0.30	0.08**
2007	0.01	0.11	0.02	0.14	0.01
2008	0.07	0.25	0.10	0.30	0.03
2009	0.09	0.29	0.12	0.33	0.03
2010	0.34	0.48	0.33	0.47	-0.01
2011	0.47	0.50	0.33	0.47	-0.14**
Observation	76		226		
**Panel B: Post-treatment**					
Duration time from diagnose to death or the last follow-up (days)	266	304	318	372	52.23
Died	0.49	0.50	0.23	0.42	-0.26**
Age of incidence	68.75	14.71	50.02	6.81	-18.00**
Breast cancer	0.35	0.48	0.45	0.50	0.10**
Cervical cancer	0.65	0.48	0.55	0.50	-0.10**
Rural area	0.59	0.49	0.64	0.48	0.06
Married but not widowed	0.77	0.42	0.99	0.08	0.22**
Peasant	0.84	0.37	0.84	0.37	0.00
Year of incidence					
2012	0.20	0.40	0.16	0.37	-0.04
2013	0.08	0.27	0.12	0.33	0.04
2014	0.25	0.43	0.12	0.33	-0.13**
2015	0.36	0.48	0.48	0.50	0.12**
2016	0.11	0.31	0.12	0.32	0.01
Observation	148		608		

Note: Significance codes

*, p<0.10

**, p<0.05

***, p<0.01.

Panel B reports the summery statistics in the post-treatment period. The duration time since the diagnosis with cancer is still longer for the control group, but the difference becomes statistically insignificant. This is mainly because majority of the patients are still alive, that means that we do not observe most people’s final life spans. In order to further investigate the difference in life span between control and treatment group, we estimate the (unconditional) Kaplan-Meier survival functions, which is reported by Fig 1. We can find that the survival functions have huge difference in both pre- and post-treatment period. A minor change in the post-treatment period is the difference in survival functions between the two groups increases a little larger, especially in the first a few years since the diagnose. In addition, in the post-treatment period, both the control and treatment groups die quicker than the pre-treatment period. *Moreover*, in the post-treatment, patients are more likely to be diagnosed at older ages, come from rural areas and take some non-farming jobs. Also, maybe more important, the patients of cervical cancer become more in the treatment group. This may be because the breast cancer is more likely be discovered by patients than cervical cancer. As a result, the screening program are more likely to discover the patients with cervical cancer who would not without the screening program. *Finally*, what we need to note is that there are more observations in the treatment group relative to the control group in the post-treatment period than in the pre-treatment. This implies that the eligible women were encouraged to receive the cancer examination by the screening program, while some of them might not receive the test if there would not be the screening program.

## Results

**Fig 2:**
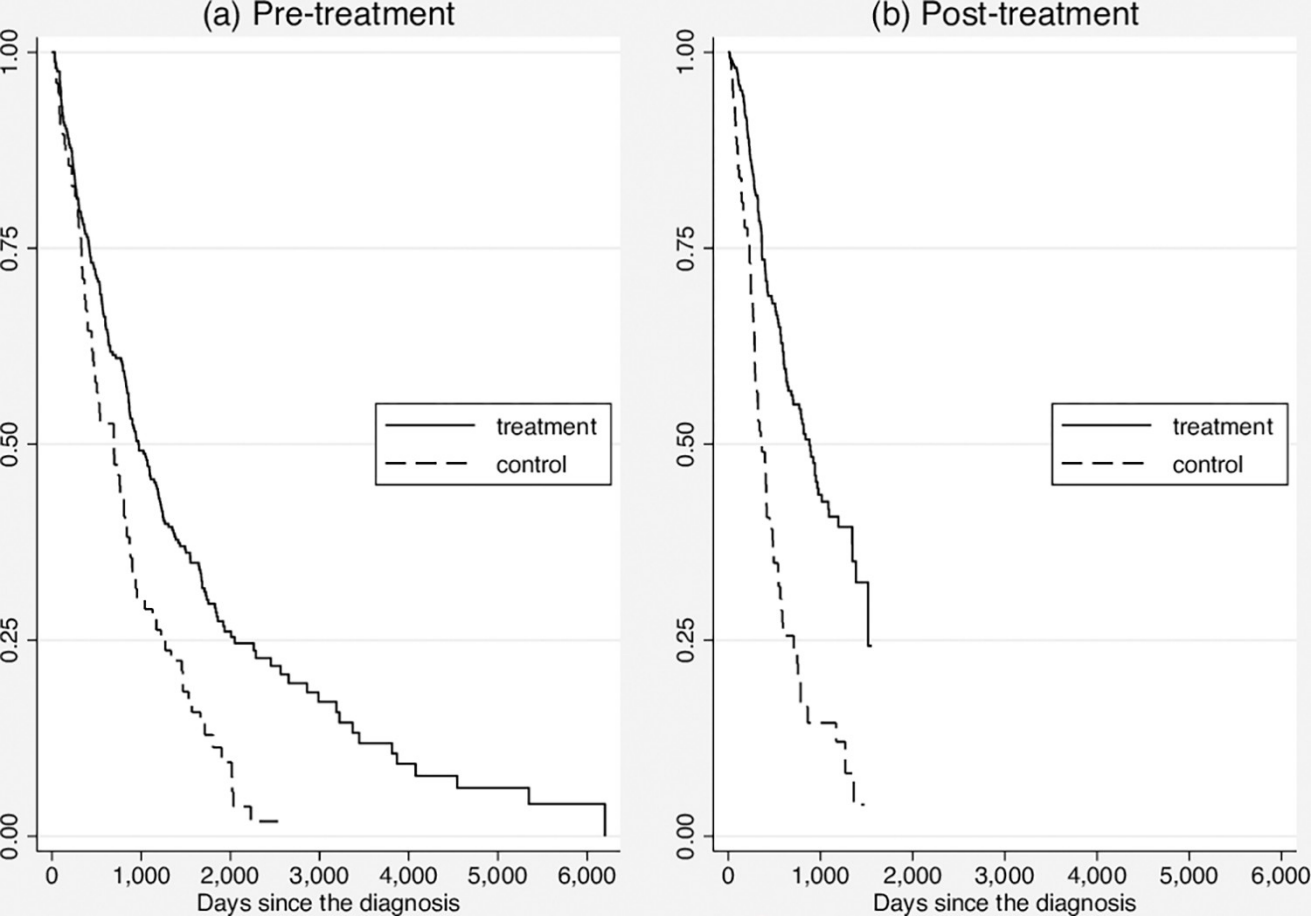
Kaplan-Meier survival estimates.

Table 2 reports results from the Cox PH DiD models, where the first two columns are for the Model M1. Column (1) presents the original estimated parameters, while Column (2) presents the corresponding effects on the hazard ratio (HR). The first row of Columns (1) and (2) suggests that ceteris paribus in the pre-treatment period the control and treatment groups have no significant difference. The second row indicates that the likelihood of dying in the post-treatment period is about 0.6 time higher compared to the pre-treatment period, which is consistent with what we have found in Fig 2. In the third row, where our primary focus is upon, the coefficient of the interaction between the dummy for post-treatment and the dummy for treatment is -0.475 which is significant at 5% level, and the effect on the HR is 0.622. These results tell that the screening program makes likelihood of the diagnosed patients dying reduces by about 40% holding all other factors fixed, which is a huge effect. *This has a strong policy implication*: *universalizing the screening program can save more people’s lives*, *or at least should be able to extend the potential patients’ life spans*. Finally, Columns (1) and (2) also *suggest female peasants are much more likely to die than the women taking non-farming jobs*. This may be related to the income, health knowledge and perception and the accessible facilities. However, because of the data limitation, we cannot further check what factors lead to the much higher likelihood of dying among peasant patients.

**Table 2:** The results of the Cox PH DiD model.

	Model M1		Model M2	
	Orig. Par.	HR	Orig. Par.	HR
VARIABLES	(1)	(2)	(3)	(4)
Treat	0.127	1.136	0.093	1.097
	(0.179)	(0.203)	(0.183)	(0.201)
POST	0.482***	1.620***		
	(0.162)	(0.263)		
POST∙Treat	-0.475**	0.622**	-0.490**	0.613**
	(0.210)	(0.131)	(0.211)	(0.129)
Years of incidence				
2007			0.411	1.509
			(0.385)	(0.581)
2008			0.728***	2.072***
			(0.264)	(0.547)
2009			1.299***	3.665***
			(0.151)	(0.555)
2010			0.915***	2.498***
			(0.212)	(0.528)
2011			1.060***	2.887***
			(0.187)	(0.540)
2012			1.420***	4.137***
			(0.264)	(1.092)
2013			1.543***	4.681***
			(0.296)	(1.387)
2014			1.580***	4.856***
			(0.262)	(1.275)
2015			1.402***	4.062***
			(0.318)	(1.293)
2016			1.193	3.296
			(1.068)	(3.521)
Age of incidence	-0.005	0.995	0.002	1.002
	(0.039)	(0.039)	(0.039)	(0.039)
Age of incidence squared	0.000	1.000	0.000	1.000
	(0.000)	(0.000)	(0.000)	(0.000)
Rural	-0.003	0.997	0.008	1.008
	(0.104)	(0.103)	(0.111)	(0.112)
Married	0.095	1.100	0.107	1.113
	(0.281)	(0.309)	(0.272)	(0.302)
Peasant	0.923***	2.516***	0.860***	2.364***
	(0.167)	(0.421)	(0.178)	(0.421)
Observations	1,058	1,058	1,058	1,058

Note: Robust standard errors in parentheses, which are clustered at the village/community level. Significance codes

*** p<0.01

** p<0.05

* p<0.1.

Columns (3) and (4) reports the estimation results of Model M2. Basically, there is no big difference compared to those for Model M1. The coefficients of dummies for years of incidence in the pre-treatment period are lower than those in the post-treatment period generally. This is consistent with the findings in Fig 1 and Model M1.

It is well known that the DiD model needs the parallel trend assumption holding in the pre-treatment period. In order to test this, we run the following regressions:

$$\lambda (t)=\lambda_{0}(t)\exp\left\{\beta_{1}{POST}_{j}+\beta_{2}{Treat}_{i}+\beta_{3}({POST}_{j}∙{Treat}_{i})+\beta_{4}D_{2010}+\beta_{5}(D_{2010}∙{Treat}_{i})+\beta_{6}D_{2011}+\beta_{7}(D_{2011}∙{Treat}_{i})+X_{ij}^{\prime }\delta +\varepsilon_{ij}\right\},$$ (M1A)

and

$$\lambda (t)=\lambda_{0}(t)\exp\left\{\sum_{j=2007}^{2016}{(\beta }_{1}^{j}∙D_{j})+\beta_{2}{Treat}_{i}+\beta_{3}(POST∙{Treat}_{i})+\beta_{5}(D_{2010}∙{Treat}_{i})+\beta_{7}(D_{2011}∙{Treat}_{i})+X_{ij}^{\prime }\delta +\varepsilon_{ij}\right\},$$ (M2A)

where *D*_2010_ and *D*_2011_ are two dummy variables indicating diagnose with cancer in 2010 and 2011, respectively. Here, because there are only very limited observations especially in the control group before 2010, the base group of incidence years for the interaction terms is set to 2006–2009. If the parallel trend assumption holds in the pre-treatment period, the coefficients *β*_5_ and *β*_7_ should be statistically insignificant.

Table 3 presents the validity tests for our Cox PH DiD model. The results in Table 3 show that adding these terms changes the results slightly, for example, the significance level of the effect of the screening program reduces a little bit. More important, the two interaction terms of D_2010_∙Treat_*i*_ and D_2011_∙Treat_*i*_ are insignificant at all. This implies that the DiD model is applicable for this research question and our previous results are credible.

**Table 3:** The validity test for the Cox PH DiD model.

	Model M1a		Model M2a	
	Orig. Par.	HR	Orig. Par.	HR
VARIABLES	(1)	(2)	(5)	(6)
Treat	0.109	1.116	0.079	1.082
	(0.216)	(0.241)	(0.202)	(0.218)
POST	0.544**	1.723**		
	(0.214)	(0.369)		
POST∙Treat	-0.469*	0.626*	-0.468*	0.626*
	(0.255)	(0.160)	(0.241)	(0.151)
D_2010_∙Treat	-0.019	0.982	-0.024	0.976
	(0.310)	(0.304)	(0.291)	(0.284)
D_2011_∙Treat	0.075	1.078	0.066	1.068
	(0.301)	(0.324)	(0.297)	(0.317)
All controls	Yes	Yes	Yes	Yes
D_2010_ and D_2011_	Yes	Yes	Yes	Yes
Dummies for all other years of incidence	No	No	Yes	Yes
Observations	1,058	1,058	1,058	1,058

Note: Robust standard errors in parentheses, which are clustered at the village/community level. All regressions control for age of incidence and its square, dummies for rural areas, marriage status and occupation. Significance codes

*** p<0.01

** p<0.05

* p<0.1.

## Robustness and placebo tests

In this section, we conduct two robustness tests and one placebo test.

First, we combine the propensity score matching and the Cox PH DiD model. Because the previous tests have shown that the parallel assumption hold in our data, the matching is not necessary, but combining the matching and the DiD model is helpful to improve the estimation precision. Panel A of Table 4 reports these matching DiD estimation results. Here, we include the dummies for the rural areas, marriage status, occupation and the interaction terms between any two of these three dummy variables, as well as the dummies for the years of incidence when calculating the propensity score. Then we choose the one-to-one matching since the stcox command in Stata does not support weight for the one-to-K matching, and there are 863 observations in the matched sample. With the matched sample, we estimate the models again. The results remain similar as in Table 2 with an exception that the magnitudes of the effect of screening program become larger.

**Table 4:** Robustness tests.

	Model M1		Model M2	
	Orig. Par.	HR	Orig. Par.	HR
VARIABLES	(1)	(2)	(3)	(4)
**Panel A: Cox PH DiD + matching**				
POST∙Treat	-1.126***	0.324***	-0.899**	0.407**
	(0.410)	(0.133)	(0.393)	(0.160)
Observations	863	863	863	863
**Panel B: Exclude the patients diagnosed in 2012**				
POST∙Treat	-0.674***	0.510***	-0.682***	0.506***
	(0.238)	(0.121)	(0.245)	(0.124)
Observations	931	931	931	931
**Panel C: Exclude the patients diagnosed before 2011**				
POST∙Treat	-0.564**	0.569**	-0.539**	0.583**
	(0.248)	(0.141)	(0.247)	(0.144)
Observation	874	874	874	874

Note: Robust standard errors in parentheses, which are clustered at the village/community level. The model specifications are exactly the same as those in Table 2. Significance codes

*** p<0.01

** p<0.05

* p<0.1.

Second, we exclude the observations in 2012. Given 2012 is the first year of the screening program was begun to implement, it is possible that some village/community doctors could not inform the potential patients. Or because of some other reasons, the screening program might not be well implemented in the first year. In this case, the magnitude of the effect of the screening program should be underestimated. Panel B of Table 4 reports these results excluding the incidence in 2012. We can find that the magnitude of the effect of screening program is larger than that in the full sample, which confirms our guess that in the first year the program might not be implemented well.

Third, China initiated the New Rural Cooperative Medical Scheme (NCMS) in 2003, and Nown County is one of eight pilot counties. By 2011, 100% of rural residents were covered by the NCMS in Nown County. In addition, China also implemented the Urban Residents Medical Scheme (URMS) in 2007 as the supplement for the old Urban Employee Medical Scheme (UEMS). By 2010, almost all urban residents can be covered by either URMS or UEMS. Given that the medical expenditure can be partially covered by one of the medical insurances, the estimated effect of the screening program may be contaminated. In order to isolate this confounding effect, we drop the observations who were diagnosed with a cancer before 2011. With this data exclusion, all the patients left in the sample should be covered by some of the medical insurance when they were diagnosed with a cancer. Thus, the reforms in the medical insurance should not affect our estimated effects of the screening program. Panel C of Table 4 reports these results, which have only trivial changes relative to our baseline results.

**Fig 3:**
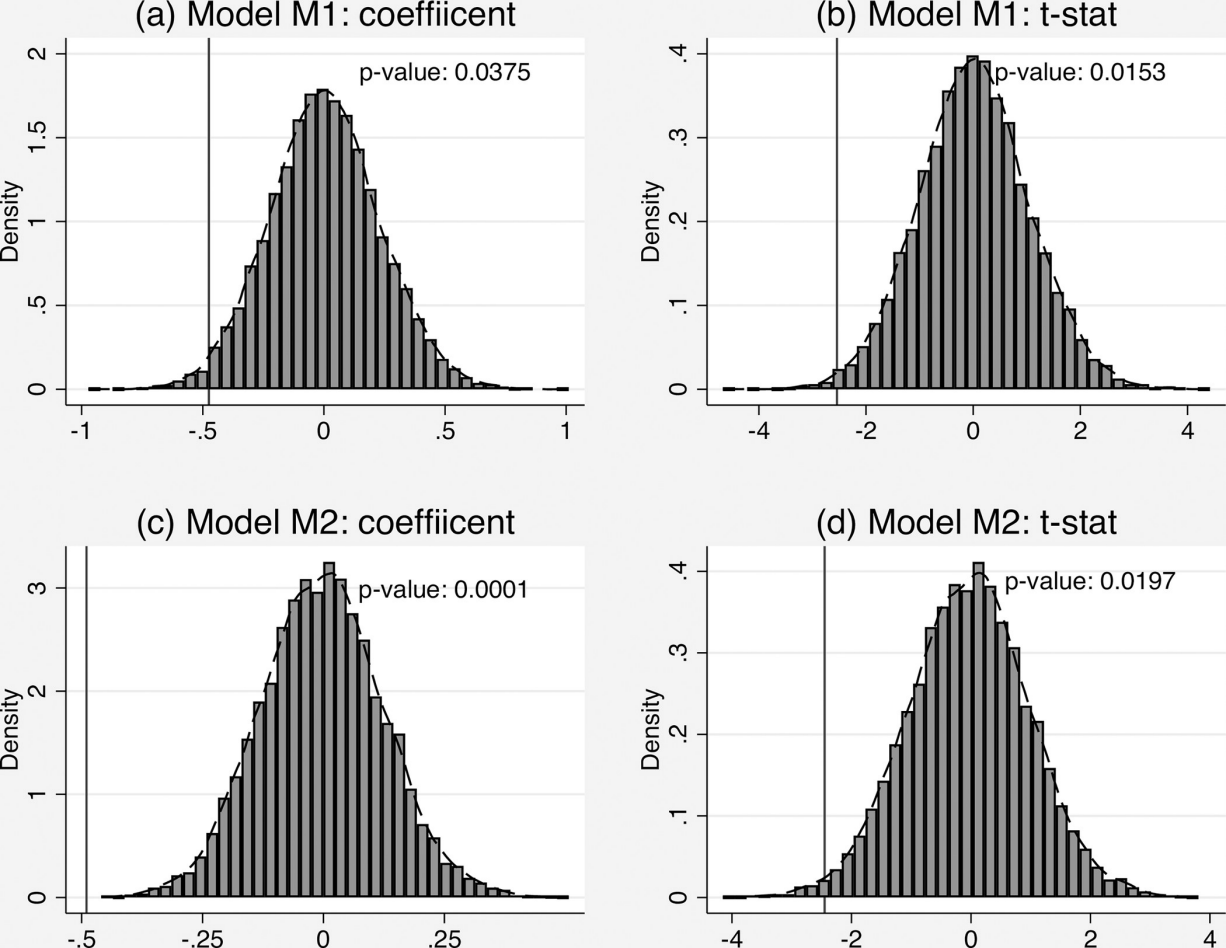
Distribution of estimated coefficient and t-statistic of (POST∙Treat) resulting from 10,000 random assignments to exposure to the cervical and breast screening program. Note: the vertical lines in the figure represent the location of the estimated coefficient and t-statistic of the actual treatment effect (the estimates for the original parameters in Models 1 and 2) within the distribution.

Finally, we make an alternative statistical inference based on placebo treatments. Precisely, we randomly assign the year of incidence to each patient without replacement. Therefore, in the same year the same number of patients receives the placebo cervical and breast cancer examination who might receive the cancer in the year, some year before or after. Because the screening program was implemented in 2012, these eligible patients are subject to a placebo treatment (exposure to the cancer screening program). We randomly draw 10,000 sets of placebo treatment assignments and estimate Models M1 and M2. Fig 3 plots the distribution of the estimated coefficients and t-statistics from the 10,000 random placebo treatments and the vertical lines represent the location of the estimated coefficients and t-statistics of the actual treatment effect within the distribution. Here we only present results for the estimate for the original parameters in Models M1 and M2. The results for the hazard rate are available upon requests. In Fig 2, we also report the share of the placebo coefficient estimates and t-statistics which are larger than the absolute value of the actual statistic. These can be viewed as the p-value approximately. In brief, these placebo tests confirm our main results.

## Heterogeneity analyses

In this section, we conduct three heterogeneity analyses to examine whether the effect of the cancer screening program differs across different groups of patients. Table 5 presents these heterogeneity analyses results.

**Table 5:** Heterogeneity analyses.

	Model M1b		Model M2b	
	Orig. Par.	HR	Orig. Par.	HR
VARIABLES	(1)	(2)	(3)	(4)
**Panel A: rural vs. urban**				
POST∙Treat	-0.541*	0.582*	-0.534*	0.586*
	(0.285)	(0.166)	(0.290)	(0.170)
POST∙Treat∙Rural	0.120	1.128	0.069	1.071
	(0.321)	(0.362)	(0.330)	(0.354)
POST∙Rural	-0.304	0.738	-0.265	0.767
	(0.242)	(0.178)	(0.243)	(0.186)
Effect on Rural patients	-0.421*	0.657*	-0.465	0.628*
	(0.245)	(0.161)	(0.246)	(0.155)
**Panel B: peasant vs. non-farming occupation**				
POST∙Treat	-1.192***	0.304***	-1.152***	0.316***
	(0.430)	(0.131)	(0.433)	(0.137)
POST∙Treat∙Peasant	0.758**	2.133**	0.711*	2.037*
	(0.385)	(0.821)	(0.387)	(0.789)
POST∙Peasant	-1.765***	0.171***	-1.672***	0.188***
	(0.447)	(0.076)	(0.444)	(0.083)
Effect on Peasant patients	-0.434**	0.648*	-0.441**	0.643**
	(0.216)	(0.140)	(0.217)	(0.139)
**Panel C: cervical cancer vs. breast cancer**				
POST∙Treat	-0.549**	0.578**	-0.567**	0.568**
	(0.240)	(0.138)	(0.241)	(0.137)
POST∙Treat∙breast	0.267	1.306	0.278	1.320
	(0.325)	(0.425)	(0.319)	(0.421)
POST∙breast	-0.252	0.778	-0.265	0.767
	(0.264)	(0.205)	(0.255)	(0.195)
Effect on breast cancer	-0.282	0.754	-0.289	0.749
	(0.301)	(0.227)	(0.294)	(0.220)
Observations	1,058	1,058	1,058	1,058

Note: Robust standard errors in parentheses, which are clustered at the village/community level. All the regressions have the same model specifications as Models M1 and M2 except that the two new interaction terms are added. Significance codes

*** p<0.01

** p<0.05

* p<0.1.

First, we investigate whether the residential address matters to the treatment effect. To this end, we add two interaction terms into Models M1 and M2: the interaction term between the dummies for post-treatment period and the rural area, and also the three-way interaction among the dummies for post-treatment period, rural area and eligibility to the treatment. We label these augmented models M1b and M2b. In these models, the interaction term of (POST∙Treat) captures the treatment effect on the patients in urban areas. The interaction term of (POST∙Treat∙Rural) captures the difference in the treatment effect between rural and urban patients. And the interaction term (POST∙Rural) captures potential change in the hazard rate of dying before and after the screening program began in the rural areas. This is the so-called difference-in-differences-in-differences (DDD) model, and the treatment effect on the rural patients can be calculated based on the sum of the coefficients of (POST∙Treat) and (POST∙Treat∙Rural). Panel A of Table 5 presents the heterogeneity analyses between rural and urban areas. It is suggested that the treatment effect is a little smaller in the rural areas but it is not statistically significant. The cancer screening program has no differential effect between rural and urban areas.

Second, we conduct the similar analysis between peasants and the patients engaging in non-farming occupations, and the results are reported by Panel B. We can find that the treatment effect is much lower for peasants: it is about a half relative to the effect on the patients taking non-farming jobs. This should be related to income, health knowledge, access to medical facilities, but again because of the data limitation we cannot test the detailed mechanisms.

Third, we investigate whether the screening program has a different effect on the patients with cervical and breast cancers. Panel C of Table 5 reports the results of this heterogeneity analysis, which suggests that screening program actually has no statistically significant effect on the patients with breast cancer. In other words, the treatment effect we identified should mainly come from the cervical cancer. This may be related to the difference between early detection screening and preventive screening. Mammography screening for breast cancer as early detection screening is designed to detect established malignant lesions while Pap‐smear screening for cervical cancer as prevention screening is applied to detect and remove precancerous lesions. Early detection screening caused the overdiagnosis, invasive treatment and psychiatric disorder (fear), which affected the final effect.

## Conclusion

The Chinese government document to promote the screening program for breast and cervical cancer is intended to reduce mortality through early detection and early treatment. Our analysis, based on Nown County data, exhibits that the screening program for breast and cervical cancer can extend the potential patients’ life spans. Our data confirms that the positive effect of the cancer screening program, which is similar with the main arguments and observations in the global academia. Our analysis, based on cancer patients of a county, can comprehensively show the effect of the screening program through the causal analysis, better than the existing literature mainly about descriptive and correlation analysis. Patterns of cancer screening and treatment in China lacked the sufficient discussion as the lack of high-quality data [39]. Our study fills in the gap over the unavailability of cancer data.

Our research observes that there was no obvious difference in the screening effect of reducing mortality between rural and urban areas. The existing literature finds that rural cervical surveillance project lacked the follow-up for those with positive results in cervical cancer screening, which influences the detection rate of cervical cancer [36], while mortality rate of cervical cancer in younger women was increasing in urban China [40]. Our study shows that the cancer screening program for rural women in China seems to increase the opportunity of early detection and early treatment, reducing the gap of medical quality between rural and urban areas.

We observe that the screening program produces better effect for those patients who taking non-farming jobs than those taking farming jobs. Rural residents normally lack the sufficient information and knowledge on cancer prevention and treatment. Women taking farming jobs means that they live in rural areas and lack the sufficient family income, health knowledge and access to the medical services, which may influence the medical treatment, consequently influences the screening effect. Sociopolitical structures of communities (culture and accessibility) are important factors in determining the cancer screening effects [41]. Socio-economic inequalities in cancer screening can be observed in opportunistic regions [42]. Culture is another important factor influencing women participating in cancer screening programs, as low screening rates may change the effect of cancer prevention. The existing study finds that old Chinese-Australian women had the lowest screening rate as they were fatalistic and thought cancer as inevitable [43]. According to Hong Kong’ survey most of women were not familiar with breast screening. Among those who knew it, time and cost are the main causes to hinder women to participate in the screening though they knew its importance [44]. Those taking non-farming jobs might have more opportunities to access the outside world and know the risk of breast and cervical cancer. Breast and cervix are two sensitive and private body parts for women in rural China, however. They normally avoid discussing such issues openly and are shamed to know the related knowledge, which might influence the screening effect of those taking farming jobs.

The difference between breast cancer screening and cervical cancer screening has also been observed. Our analysis exhibited that the effect should be mainly attributed to the cervical cancer screening rather than breast cancer screening. This discrepancy of the screening effect is closely related to the difference between early detection screening and preventive screening. The breast cancer screening as the early detective screening intends to reduce the mortality rather than cancer incidence, while the cervical cancer screening as the preventive screening is designed to detect and remove precursors of cancer rather than established malignant lesions, which can contribute to achieving the reduction in cancer mortality through the reduction of the incidence [32]. The reduction in breast cancer mortality through screening predominantly relied upon improved systemic therapy, which may bring overdiagnosis and harm [32, 45]. Another reason may be the cancer fear. Not all breast cancer is incurable or fatal, psychiatric disorder caused by early detection may influence the effect [31]. More seriously, the decision whether to preserve the breast has the fundamental effect on the quality of life, which is different from the cervical cancer. Besides, the existing data on the effect of cervical preventive screening tests is scarcely available [24, 46]. Our research fills in this gap.

However, our research also has limitations. Firstly, our variables of data are limited, which lacks the opportunity to explore the underlying reasons for the screening effect. Secondly, we only have one county data, which may not exhibit the whole picture of China in the cancer screening, since different places may have different patterns. However, it still exhibits the basic characteristics and trend to some extent, particularly in central and south China. It is necessary and valuable to have more studies like this, which will contribute to exhibiting the national picture.

In order to make breast and cervical cancer screening more effective, a lot of measures are necessary to be adopted. First, more efforts and resources should be invested to improve the effective and efficiency of breast and cervical cancer screening program. Screening funding should be increased to extend the coverage of breast and cervical cancer screening and to train medical staff to improve their capacities and skills in screening operations. Sufficient and necessary funding is the basic requirement for the effective screening result. In addition, the Chinese government needs to improve the medical care insurance to cover more cancer treatment expenses. Secondly, it is necessary to differentiate the breast cancer screening from the cervical cancer screening. The government should redesign the cancer screening program for women and set different goals for breast and cervical cancer screening in order to better realize the difference between early detection screening and preventive screening to reduce overdiagnosis and harm [32]. Third, to raise the awareness of breast and cervical cancer and let rural residents know more health knowledge are also important measures to reduce the incidence [47, 48], since they lacked the basic health knowledge [49]. Education on this knowledge can empower rural women to know the cancer risk, actively participate in the screening program and emphasize more on the daily health care. With these further actions, the breast and cervical cancer incidence and mortality rate will decline.

## Supporting information

S1 Appendix (DTA)
